# Composites Based on Eucalyptus Nitens Leaves and Natural Rubber as a Valuable Alternative for the Development of Elastomeric Materials with Low Microbiological Impact

**DOI:** 10.3390/polym16152215

**Published:** 2024-08-03

**Authors:** Héctor Aguilar-Bolados, Natacha Rosales-Charlin, Claudia Pérez-Manríquez, Solange Torres-Galan, Mohamed Dahrouch, Raquel Verdejo, Marianella Hernández Santana, Jose Becerra

**Affiliations:** 1 Departamento de Polímeros, Facultad de Ciencias Químicas, Universidad de Concepción, Concepcion 3349001, Chile; nrosales2019@udec.cl; 2 Departamento de Fitoquímica, Facultad de Ciencias Naturales y Oceanográficas, Universidad de Concepción, Concepcion 3349001, Chile; 3 Departamento de Química Orgánica, Facultad de Ciencias Químicas, Universidad de Concepción, Concepcion 3349001, Chile; mdahrouch@udec.cl; 4 Instituto de Ciencia y Tecnología de Polímeros (ICTP), CSIC, Juan de la Cierva, 28006 Madrid, Spainmarherna@ictp.csic.es (M.H.S.)

**Keywords:** Natural Rubber, Eucalyptus nitens, mechanical properties

## Abstract

The forest industry produces several low-value by-products, such as bark, sawdust, limbs, and leaves, that are not ultimately disposed of and remain in the forests and sawmill facilities. Among these by-products are leaves, which contain not only cellulose fibers and lignin but also essential oils such as terpenes. These are biosynthesized in a similar way as cis-1,4-polyisoprene. In this context, this work evaluates the use of screened and unscreened dried Eucalyptus nitens leaves in natural rubber. Among the most relevant results of this work is a significant increase in mechanical properties, such as tensile strength and elongation at break, reaching values of 9.45 MPa and 649% of tensile strength and elongation at break, respectively, for a sample of natural rubber containing sieved dried leaves of Eucalyptus nitens. In addition, it is observed that the content of this vegetable filler allows for inhibiting the antibacterial effect of vulcanized rubber against several bacteria, such as Bacillus subtilis, Staphylococcus aureus, Escherichia coli K 12, Escherichia coli FT 17 and Pseudomonas fluorescens. These results are promising because they not only add value to a by-product of the forestry industry, improving the mechanical properties of natural rubber from a sustainable approach but also increase the affinity of rubber with bacterial microorganisms that may play a role in certain ecosystems.

## 1. Introduction

Natural rubber (NR) is a complex material with unique properties, such as elasticity and flexibility, which make it challenging to replace. In addition, it is also a sustainable material, as it is not derived from fossil sources. In fact, natural rubber could be considered a carbon-positive material since *cis*-1,4-polyisoprene is mainly derived from the *Hevea brasiliensis* tree and produced by a metabolic pathway called Rubber-transferase [1].

One opportunity for NR compounds to be included in the circular economy approach is to improve their sustainability, as the NR compounds must be vulcanized and fillers added to achieve higher mechanical performance. This vulcanization process imparts several properties to natural rubber-based compounds, including resistance to biodegradation. In addition, materials used as fillers, such as carbon black or silica, have a negative carbon footprint, which adds to the environmental impact of these rubber compounds [1,2,3]. 

Due to the new challenges of our society, new trends have emerged, such as the circular economy, eco-design concepts that promote a sustainable approach, prioritizing the development of technologies that have a lower environmental impact based on the 10R concepts, and a reduced carbon footprint [4]. In this context, there is growing interest in sustainable materials that can be used as fillers in NR. Among the materials used as fillers, forest by-products stand out, mainly leaves and branches from monocultures, such as Eucalyptus nitens [4]. In addition, this lignocellulosic material possesses essential oils that have a biological role based on the semiochemical properties of this species, which are compounds or mixtures released by an organism that affect the behavior of other individuals [5].

Eucalyptus species that are more abundant are Eucalyptus globulus and Eucalyptus nitens. Eucalyptus globulus is a widely utilized medicinal plant. It contains bioactive compounds, including antioxidants, antimicrobials, and phytoremediators [6,7,8]. In addition, it has a wide range of biological activities, including antimicrobial, fungicidal, insecticidal, herbicidal, and nematocidal properties [9,10,11]. Its essential oil can also be used as an herbicide and food preservative [11,12]. Its use is significant for reducing pest resistance and minimizing the environmental impact of synthetic pesticides. In particular, compounds such as 1,8-cineole (eucalyptol) are promising candidates for the development of new pharmaceuticals and agrochemicals [13]. 

On the other hand, various materials obtained from vegetable sources have been used as fillers in natural rubber to obtain composites. This approach includes cellulose [14,15,16], sawdust [17,18], and others. In general, the key challenge is to increase the affinity of the filler for the polymer matrix. It is also known that leaves from plants containing traces of essential oils have a greater affinity for natural rubber. This affinity is due to the fact that essential oils are mainly composed of terpenes. In this context, this work focused on obtaining materials based on NR and sifted or unsifted *Eucalyptus nitens* leaves to demonstrate the affinity between the filler and natural rubber. The aim was to obtain rubber materials and examine their mechanical and morphological properties, as well as their resistance to microorganisms such as bacteria. To the best of our knowledge, this type of material is tested for the first time to obtain composites, and interesting results have been obtained to conceptualize new insights with a sustainable approach.

## 2. Materials and Methods

### 2.1. Materials

The materials used to prepare the composite specimens were natural rubber (NR) (SGR-10) obtained from Química Miralles S.A. Industry (Quilicura, Chile), and zinc oxide (ZnO), stearic acid (SA), powdered sulfur (S), N-cyclohexyl-2-mercaptobenzotiazole (MBTS), tetramethylthiuram disulfide (TMTD), which were were purchased from Sigma Aldrich (St. Louis, MO, USA), and all reagents were of ACS grade (≥95%). 

### 2.2. Preparation of Lignocellulosic Fillers

Juvenile leaves of Eucalyptus nitens were collected from the “*La Cantera y el Guindo*” located in Concepción, Bio-Bío region, Chile. They were then dried in an oven at 45 °C for 14 days. The dried leaves were ground in an automatic laboratory mill PRO-02; half of the ground leaves were sifted through a #50 mesh sieve, and the other half was left unsifted.

### 2.3. Determination of Essential Oil Content in Filler

A yield analysis of the 3 types of samples, dry leaves (DL), sifted leaves (SF), and unsifted leaves (USF), was performed using the same conditions of water, temperature, time, and amount of sample by using the method based on hydrodistillation with a Clevenger apparatus. This procedure consisted of a hydrodistillation that allowed to obtain the essential oils present in the samples. The principle of this separation technique is that the steam generated entrains the essential oils present in the samples studied; this oil is then condensed and collected for subsequent analysis. For this purpose, Eucalyptus nitens leaf-based material was placed in a 3-L balloon connected to a cooling column and an essence extractor (Clevenger apparatus). This mixture was allowed to boil until the volume of extracted essential oils remained stable, which took about 3 h. At the end of the extraction period, the aqueous solution was immediately collected in a decanting funnel wrapped in aluminum and allowed to stand for a few minutes. The hydrolate, which corresponds to an aqueous solution of the hydro-soluble compounds of *Eucalyptus nitens*, was then separated from the essential oil and transferred to a clean vial. Sodium sulfate was added to the vial to remove excess water. The amount of essential oil was then measured with a 10 mL graduated cylinder and stored in a new vial with a clean cap, wrapped in aluminum, and stored at room temperature.

GC-MS analysis of the essential oils was performed using a gas chromatograph (Agilent 7890A, Santa Clara, CA, USA) with a splitless injector (250 °C) and a mass detector (Agilent 5975C). An HP-5MS capillary column (30 m × 0.25 mm × 0.25 µm) and helium gas (constant flow rate 1 mL/min) were used for separation. The temperature program was 5 min at 100 °C, 100–275 °C at 13 °C/min and 32 min at 275 °C. The detection range was m/z 50–550. The injector temperature was set to 250 °C. The components were identified by comparing the mass spectra with records from NIST 17 (NIST/EPA/NIH Mass Spectral Library 2017), and by comparing the spectra obtained with those reported in the literature [19].

### 2.4. Composites Preparation

The NR rubber composites were prepared using a ZL-3018 two-roll mill (Zhongli Instrument Technology Co., Ltd., Dongguan, China) according to the formulations described in Table 1 at room temperature for 20 min. The mixing consisted of dispersing first the activators (ZnO and stearic acid) into the NR rubber, then incorporating the filler (USF or SF), followed by the addition of accelerators (MBTS and TMTD) and sulfur (S). 

### 2.5. Characterization

The characteristics of the vulcanization systems, minimum torque (*M_L_*), maximum torque (*M_H_*) scorch time (*t_s_*_2_), and vulcanization time (*t*_90_), were determined using a ZL-3001 moving matrix rheometer (Zhongli Instrument Technology Co. Ltd., China) for 30 min at 160 °C. Vulcanization of the composites was performed using a ZL-3022 laboratory hydraulic press (Zhongli Instrument Technology Co. Ltd., Dongguan, China) for 15 min at 120 Kg/cm^2^ pressure and 180 °C [20]. The hardness was determined using a rectangular die of 11.5 × 13 × 0.2 cm according to ASTM 2240-15.

The stress-strain test was performed using a Shimadzu EZ-X L 200 V instrument with a load cell of 500 N at 100 mm/min, according to ASTM D 412. Resilience (ASTM D2632-15, West Conshohocken, PA, USA) and hardness Shore A (ASTM D2240-15) were also determined using tester equipment provided by Zhongli Instrument Technology Co., Ltd., (Dongguan, China). The morphology of the samples was examined by scanning electron microscopy (SEM) using a Zeiss (Oberkocken, Germany) scanning electron microscope, model Gemini SEM 360, equipped with an Oxford Instrument (Abingdon, UK) EDS detector. The samples were then covered with an ultrathin gold (Au) film. The acceleration voltage was 10 kV.

A model FT/IR-4X spectrometer, Jasco (Tokyo, Japan), was used for infrared analysis in attenuated total reflection mode (FTIR-ATR). Spectra were recorded in the range of 500 to 4000 cm^−1^. Dynamic mechanical analysis (DMA) was performed using a dynamic mechanical analyzer MCR 702e MultiDrive Anton Paar (Graz, Austria). 

### 2.6. Biological Tests

A 6 mm punch was used to cut and autoclave 150 mm Grade 1 filter papers and replicates of the 7 samples, as well as uncured rubber samples. The 6 mm filter papers were impregnated with 8 µL of *Eucalyptus nitens* essential oil mixed 50% with ethanol, in addition to papers impregnated with ethanol only, and allowed to dry in a hood near a burner for 15 min. While the dilutions of bacteria were being prepared, the test tubes with the growth of bacterial colonies of *Pseudomonas fluorescens*, *Bacillus subtilis*, *Staphylococcus aureus*, *Escherichia coli* K-12, and *Escherichia coli* FT-17 in distilled water were taken and transferred with a Pasteur pipette to another test tube containing autoclaved distilled water until the appropriate turbidity pattern was obtained to be compared with the 0.5 McFarland’s standard tube. Using seeding handles, the culture of each bacterium was spread over the whole of the plates containing Mueller-Hinton agar, and then the rubber samples with lignocellulosic filler and the already-dried filter papers were labeled and placed. *Pseudomona fluorescens*, *Bacillus subtilis,* and *Staphylococcus aureus* were incubated at 30 °C and the two *E. coli* strains at 37 °C. Measurements were made at the inhibition halos presented on the plates after 24 h

## 3. Results and Discussion

Table 2 shows the compounds identified by gas chromatography of the essential oils extracted from the unsifted (USF) and sifted (SF) Eucalyptus nitens dry-leaf-based powders. This table suggests a probable structure, approximate molecular weight, compound type, and area percentage of the major species detected. It is possible to observe that during the processing of the dry leaf powder, the content of the species changes; those with lower molar mass and lower boiling point tend to decrease their content, while those with higher boiling points tend to increase their content (Table 2). This could be explained by the fact that the grinding and sieving processes facilitate the evaporation of more volatile compounds. This is evident, for example, for 1R-α-pinene and eucalyptol (cineol), which is a volatile monoterpene, compared to argomadendrene and globulol, which are sesquiterpenes of higher molecular weight and less volatile [21,22] The identified compounds mostly have structures related to the cross-linking process, mainly because they have double bonds.

**Figure 1:**
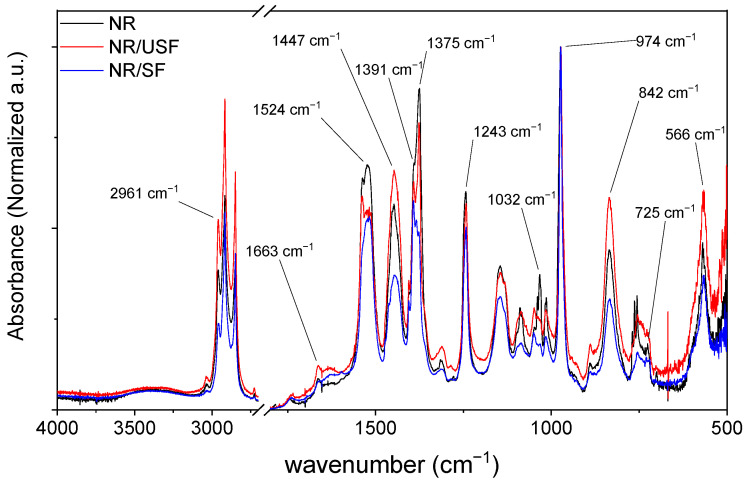
FTIR spectra of NR composites containing USF and SF.

Figure 1 shows the FTIR spectra of the NR samples and those containing sifted (SF) and unsifted (USF) vegetable fiber-based fillers. As can be seen, all spectra exhibit dominant signals characteristic of NR, such as those observed at 2961 cm^−1^ and 1524 cm^−1,^ corresponding to CH stretching and C-C vibration, respectively. In addition, absorption bands were observed at 1447 cm^−1^ and 1375 cm^−1^, which were associated with CH_2_ and CH_3_ deformations, respectively. Meanwhile the signals at 974 cm^−1^, 842 cm^−1^ and 560 cm^−1^ correspond to out-of-plane bending [23]. It is interesting to note that the main changes observed in the samples are subtle but associated with stretching of the C=S groups, and the characteristic absorption bands for this group are those recorded at 1391 cm^−1^, 1032 cm^−1,^ and 725 cm^−1^ [24,25]. The band observed at 1032 cm^−1^ decreased significantly in samples containing fillers, which could be associated with the interaction between the filler and the polymer matrix. 

### 3.1. Curing Curves and Vulcanization Parameters of NR-Based Composites

The values of minimum torque (*M_L_*), maximum torque (*M_H_*), scorch time 2 (*t_s_*_2_), and optimum vulcanization time (*t*_90_) of the NR composites are described in Table 3. It can be observed that the content of sifted and unsifted eucalyptus dry leaf powder has no significant effect on the *M_H_* recorded. Similarly, it is pertinent to mention that the *M_L_* slightly increased, which could be attributed to the effect of the vegetable filler content on the torque, which, by the hydrodynamic effect, gives more stiffness to the rubber system. However, the t90 also increases as the filler content increases, while the MH is not significant, being lower than the MH of unfilled NR. This suggests a slowing down of the vulcanization rate, probably due to the effect of the filler, which inhibits the diffusion of curing agents [26]. In addition, its chemical nature could probably play a role in such a process [1]. In fact, it is well known that vegetable fillers are made of wood, their pH is below 5.0, and they tend to be hygroscopic, properties that could interfere with the vulcanization process [27,28] In addition, since wood is mainly composed of cellulose and lignin, and the latter is used as a sorbent material, it is likely that the curing agent will be adsorbed by the filler, hindering the vulcanization process [29].

### 3.2. Mechanical Properties, Hardness and Resilience of NR Composites

Table 4 shows the modulus values at different elongations (M100, M300, and M500), tensile strength (TS), and elongation at break (EB) of the rubber composites. The unfilled rubber sample has a maximum elongation of only 145%, and its M100 is 1.26 MPa. However, when the filler was added, the maximum elongation increased and reached 699% for the sample containing 40 phr of sifted leaf powder. Similarly, the unsifted samples showed an increase in elongation. This indicates that this lignocellulosic material, which contains traces of terpenes, tends to be compatible with NR and promotes the cohesion of the polymer chains through discrete cross-linking. It should be noted that monoterpenes and sesquiterpenes originate from a metabolic pathway related to that of NR and are structurally similar in polarity [30]. Moreover, the higher elongation recorded for the compounds containing sifted fibers, smaller than that of unsifted fibers, allows the system to present a homogeneous character in particle size distribution, which also contributes to achieving a homogeneous distribution and higher phase continuity than that of those composites containing unsifted filler [31]. Table 4 also shows the hardness and resilience values of the rubber compounds. Hardness tends to show a marginal increase with the content of sifted and unsifted EAF powder, a behavior attributed to the presence of filler in the composite composition of a harder material, such as the vegetable filler used in this research, than that of NR. Meanwhile, resilience tends to gradually decrease as the filler content increases. The filler appears to have a discrete effect on the viscoelastic properties of NR. The loss of resilience as a result of increasing the filler content is attributed to the fact that the filler occupies the free volume of the rubber, which hinders the movement of the rubber chains. 

**Figure 2:**
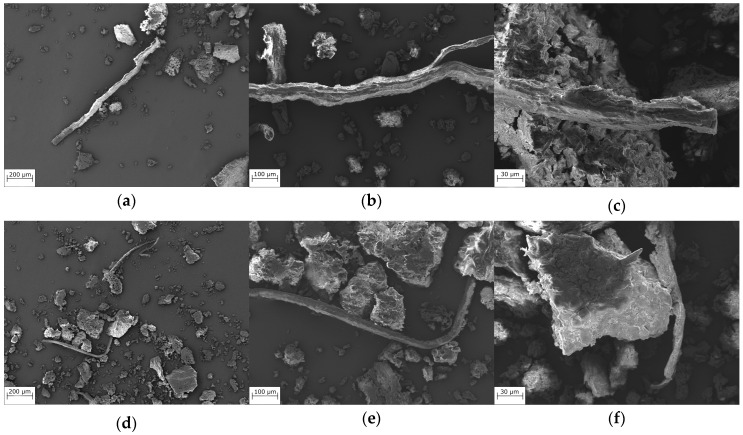
SEM images of USF (**a**–**c**) and SF (**d**–**f**).

Figure 2 shows the SEM images of the unsifted and sifted fillers. As can be seen, in the case of the unsifted fillers, the fibers have a wide size distribution, with some of them having a high aspect ratio. In contrast, the sifted filler had a more homogeneous size distribution, demonstrating the effectiveness of the milling and sieving processes.

**Figure 3:**
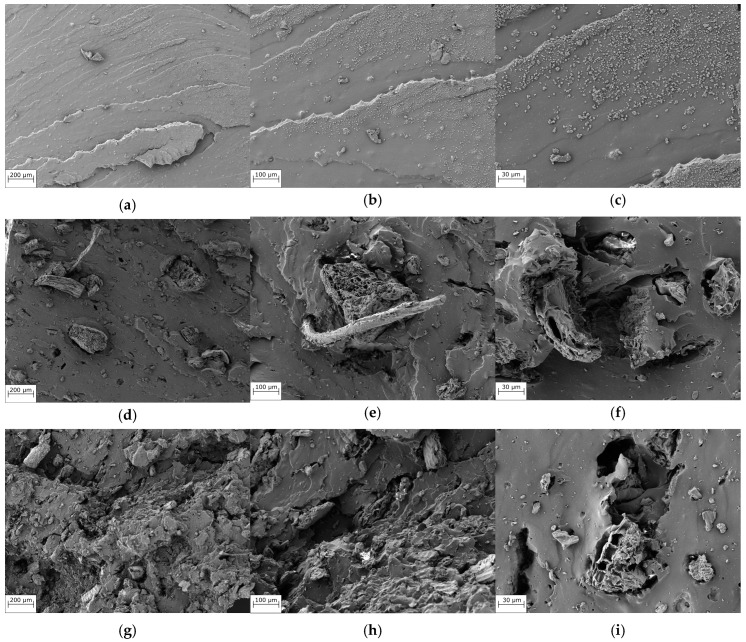
SEM images of NR (**a**–**c**), NR/USF (**d**–**f**), and NR/SF (**g**–**i**).

Figure 3 shows the SEM micrographs of the fracture cross-section of NR and NR composite specimens containing 20 phr sifted and unsifted fillers, as well as composites that showed significant differences in mechanical properties. The phase homogeneity of rubber is altered by the presence of the filler material. As seen in Figure 3d–f, the samples containing USF show particles and fibers randomly distributed in the rubber matrix, while those containing sifted filler (Figure 3g–i) show particles of comparable diameters distributed in the polymer matrix. It is worth mentioning that although the phase continuity is modified by the continuity of the filler, it can be inferred that the filler has a certain affinity for the polymeric phase. This is supported by the shape of the analyzed zone, where it was observed that the filler maintained cohesion with the polymer matrix at the time of fracture (Figure 3f,i). This could explain the significant improvement in the mechanical properties of composites containing vegetable fillers compared to NR.

### 3.3. Dynamic Mechanical Analysis of Rubber Composites

**Figure 4:**
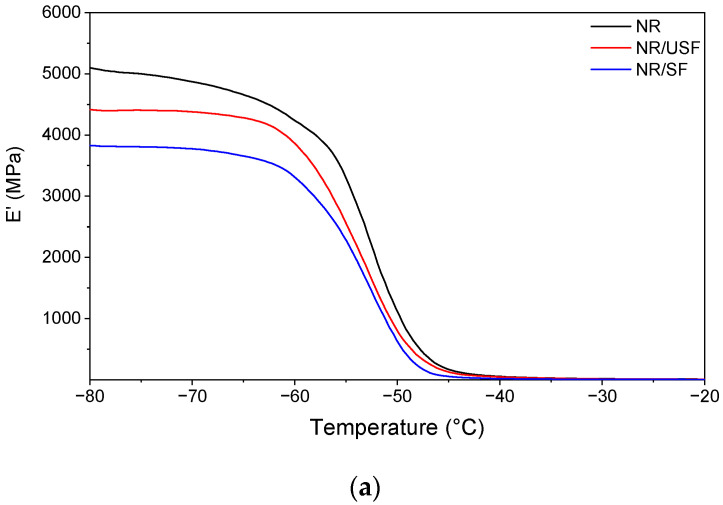

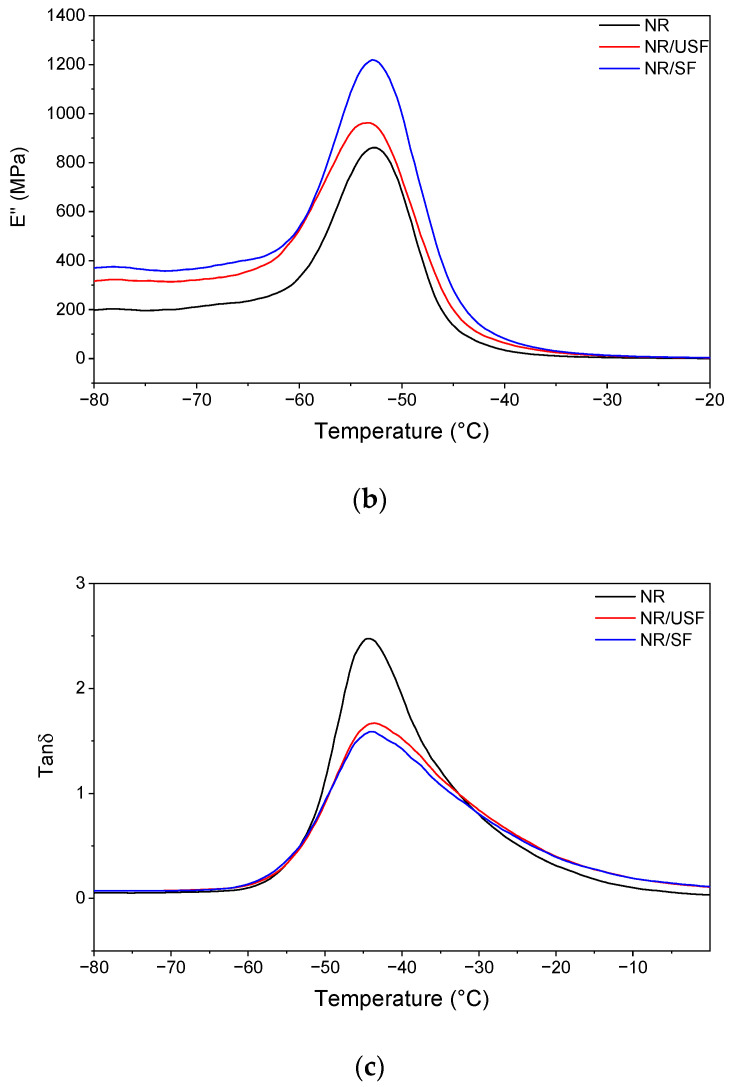
Dynamic mechanical analysis of NR, NR/USF, and NR/SF composites: (**a**) storage modulus (E′), (**b**) loss modulus (E″), and (**c**) loss factor (tanδ).

Figure 4 shows the storage, loss modulus, and loss factor of the NR-based samples and filler-containing composites obtained by DMA.

In Figure 4a, it is evident that the storage modulus of the samples with the vegetable filler is compared to that of NR. This increase can be attributed to the reinforcing role of the fibers and their specific compatibility with the matrix. Furthermore, the higher increase observed in the sample containing the sifted filler provides further evidence of this compatibility. This increase is likely due to the greater regularity in the size and smaller size distribution of the filler material, which facilitates the interaction between the polymer and the filler.

The loss moduli (Figure 4b) of the composites with fillers at low temperatures are comparable to and higher than those of the unfilled NR. However, there was a significant difference between the loss moduli of the composites with sifted and unsifted fillers. This suggests that the morphology and size distribution of the fillers influence stress dissipation. It is important to note that the curves reach their maxima at approximately −53 °C, which corresponds to the glass transition temperature (Tg) of these materials.

### 3.4. Study of Microorganism Affinity to Natural Rubber-Based Materials

Table 5 displays the average inhibition radius values of vulcanized NR for the bacteria *Bacillus subtilis*, *Staphylococcus aureus*, *Escherichia coli* K 12, *Escherichia coli* FT 17, and *Pseudomonas fluorescens*. It is evident that rubber additives, such as accelerator systems and activators, inhibit bacterial growth, indicating their toxicity. The inhibition is more pronounced for *Staphylococcus aureus,* while *Pseudomonas fluorescens* is less affected by these additives. This inhibitory effect contrasts with the behavior of the studied species against pristine unvulcanized NR, where proliferation occurs without disturbance and no inhibition halo is observed. It is worth noting that *Pseudomonas* has been used for the biological degradation of NR [32]. 

**Figure 5:**
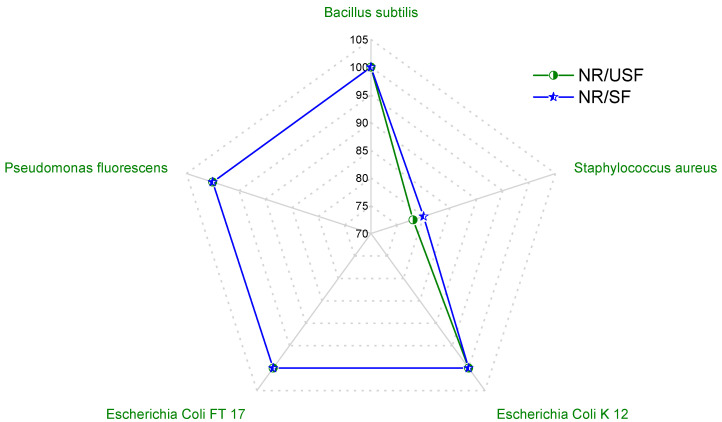
Radial plot showing the MA index for NR/USF and NR/SF, both containing 40 phr of filler.

In order to determine the level of inhibition caused by the filler, an equation has been developed to compare the inhibition diameters of vulcanized rubber with those of rubber containing the filler. This comparison is expressed as the material affinity index for microorganisms (*MA index*), which is defined by Equation (1).

(1) $$MA\;index=\frac{\left(D_{0}-D_{c}\right)}{D_{0}}\times 100$$

where *D*_0_ is the inhibition diameter of the unfilled material, and *D_c_* is the inhibition diameter of the composite. This index was estimated for each of the microorganisms mentioned in Table 5. It can be observed in Figure 5 that the *MA* indexes of the NR-based composite containing unsifted and sifted fillers showed more affinity of the microorganism to this type of material. This indicates that the filler content produces a change in the behavior of the microorganisms toward the composite, reaching maximum proliferation. This indicates that the antimicrobial nature of vulcanized natural rubber decreases, which is probably promoted by the presence of biomolecules such as polysaccharides, which act as carbon sources, favoring bacterial growth [33]. 

Based on the results obtained, it was found that although the microorganisms studied were pathogenic, a variety of microorganisms grew in the presence of the leaf-based filler. This indicates that the filler provides a carbon source, such as a polysaccharide (cellulose), which promotes the proliferation of bacteria. Since Eucalyptus nitens leaves are a complex forestry by-product containing cellulose, lignin, and essential oil, more may not only provide a better affinity for microorganisms but also improve the performance of natural rubber composites. This aspect is interesting because this material can be used in applications where bacterial proliferation is required or minimal impact on bacterial colonies is required, such as a specific ecosystem. 

## 4. Conclusions

Composites based on natural rubber and a filler derived from Eucalyptus nitens leaves were prepared. A discrete slowing down of the cross-linking process and a decrease in the maximum modulus were observed, which can be attributed to the chemical structure of the plant filler as well as to the adsorption of the cross-linking agents. The addition of this filler to rubber promoted an increase in the mechanical properties, significantly increasing the elongation at break and tensile strength. It was demonstrated that the presence of a filler prepared from dry shredded and sifted leaves promoted a greater increase in mechanical properties than the unsifted filler. On the other hand, the vegetable filler content facilitated bacterial proliferation, increasing the affinity index of the material for microorganisms such as Bacillus subtilis, Staphylococcus aureus, Escherichia coli K 12, Escherichia coli FT 17, and Pseudomonas fluorescens. This higher affinity was attributed to the chemical nature of the filler, which is composed of macromolecules that would act as a carbon source for bacteria. These results are interesting because materials that have a higher affinity for microorganisms, such as bacteria, may have a lower environmental impact on various ecosystems.

## Figures and Tables

**Table 1:** Formulation of composites based on NR.

Ingredient	NR	NR/USF10	NR/USF20	NR/USF40	NR/SF10	NR/SF20	NR/SF40
NR	100	100	100	100	100	100	100
ZnO	5	5	5	5	5	5	5
SA	2	2	2	2	2	2	2
MBTS	2	2	2	2	2	2	2
TMTD	1	1	1	1	1	1	1
USF	-	10	20	40	-	-	-
SF	-	-	-	-	10	20	40
S	2	2	2	2	2	2	2

**Table 2:** Main compounds of the five species at major concentrations in DL, USF, and SF determined by gas chromatography using a qualification score, QUAL, ≥90%.

Compound Name	Boiling Point (°C)	Molecular Formula and Molecular Weight (Da)	Percentage Area (%)		
			DL	USF	SF
1R-α-Pinene	155	C_10_H_16_, 136	21.1	8.96	5.17
Eucalyptol (Cineol)	177	C_10_H_18_O, 154	56.93	38.75	37.58
α-Terpineol acetate	219	C_12_H_20_O_2_, 196	5.39	5.56	6.21
Aroma-dendrene	258	C_15_H_24_, 204	1.6	14.84	15.77
Globulol	283	C_15_H_26_, 222	5.2	9.23	10.04

**Table 3:** Curing data of the NR composites.

Sample	*t_s_*_2_ (min:s)	*t*_90_ (min:s)	*M_L_* (Nm)	*M_H_* (Nm)
NR	0:55 ± 0:01	1:26 ± 0:08	0.043 ± 0.001	0.886 ± 0.068
NR/USF10	0:59 ± 0:01	2:18 ± 0:13	0.059 ± 0.001	0.800 ± 0.001
NR/USF20	1:09 ± 0:01	2:09 ± 0:08	0.067 ± 0.005	0.852 ± 0.036
NR/USF40	1:15 ± 0:04	2:07 ± 0:03	0.088 ± 0:003	0.821 ± 0.033
NR/SF10	1:13 ± 0:03	2:04 ± 0:07	0.070 ± 0.001	0.774 ± 0.063
NR/SF20	1:16 ± 0:06	2:14 ± 0:08	0.090 ± 0.007	0.780 ± 0.039
NR/SF40	1:17 ± 0:02	2:56 ± 0:20	0.101 ± 0.000	0.857 ± 0.001

**Table 4:** Mechanical properties of NR composites consisting of moduli at 100% (M100), 200% (M200), and 300% (M300) of elongation, tensile strength (TS), elongation at break (EB), Shore A Hardness, and resilience.

Sample	M100 (MPa)	M200 (MPa)	M300 (MPa)	TS (MPa)	EB (%)	Shore A Hardness	Resilience (%)
NR	1.26 ± 0.01			1.71 ± 0.039	145 ± 3	41.6 ± 0.4	74.1 ± 0.9
NR/USF10	1.42 ± 0.08	2.40 ± 0.12		2.84 ± 0.045	240 ± 14	43.6 ± 1.0	69.7 ± 0.5
NR/USF20	1.56 ± 0.072	2.69 ± 0.21		2.69 ± 0.21	200 ± 6	46.2 ± 0.3	68.3 ± 0.3
NR/USF40	1.16 ± 0.04	1.81 ± 0.06	2.52 ± 0.083	6.42 ± 0.18	626 ± 21	43.2 ± 1.0	60.7 ± 0.5
NR/SF10	1.40 ± 0.029			1.98 ± 0.13	170 ± 8	42.7 ± 0.8	70.3 ± 0.9
NR/SF20	1.54 ± 0.03	2.48 ± 0.03	3.55 ± 0.03	11.98 ± 0.81	707 ± 41	44.0 ± 0.5	67.8 ± 0.9
NR/SF40	1.53 ± 0.06	2.44 ± 0.08	3.35 ± 0.10	9.45 ± 0.29	699 ± 25	44.1 ± 0.5	63.5 ± 4.0

**Table 5:** Inhibition diameters of NR-based materials.

	Inhibition Diameter (cm)			
	Vulcanized NR	Unvulcanized NR	NR/USF	NR/SF
*Bacillus subtilis*	2.65	0	0	0
*Staphylococcus aureus*	3.10		0.67	0.62
*Escherichia Coli* K 12	1.22		0	0
*Escherichia Coli* FT 17	1.22		0	0
*Pseudomonas fluorescens*	0.623		0	0

## Data Availability

The data presented in this study are available on request from the corresponding author.
